# Supplementary material for: A hollow mesoporous carbon from metal-organic framework for robust adsorbability of ibuprofen drug in water
Source: R Soc Open Sci. 2019 May 22;6(5):190058. doi: 10.1098/rsos.190058 (PMC6549975; doi:10.1098/rsos.190058)
Supplement: Supplementary information [file rsos190058supp1.docx]

# Supplementary information

**A hollow mesoporous carbon from metal-organic framework for robust absorbability of ibuprofen drug in water**

Thuan Van Tran^1^, Duyen Thi Cam Nguyen^2^, Hanh Thi Ngoc Le^3^, Oanh Thi Kim Nguyen^1^, Vinh Huu Nguyen^1^, Thuong Thi Nguyen^1^, Long Giang Bach^1^, Trinh Duy Nguyen^1^*

**Affiliation**

^1^ *NTT Hi–Tech Institute, Nguyen Tat Thanh University, 298–300A Nguyen Tat Thanh, Ward 13, District 4, Ho Chi Minh City 700000, Vietnam.*

^2^ *Department of Pharmacy,* *Nguyen Tat Thanh University, 298–300A Nguyen Tat Thanh, Ward 13, District 4, Ho Chi Minh City 700000, Vietnam.*

^3^ *Institute of Hygiene and Public Health, 159 Hung Phu, Ward 8, District 8, Ho Chi Minh City 700000, Vietnam.*

**Correspondence**

* To whom correspondence should be addressed.

T.D. Nguyen (Email: [ndtrinh@ntt.edu.vn](mailto:ndtrinh@ntt.edu.vn))

Tel: (+84)-028-3941-1211 Fax: (+84)-028-39-404-759

## **Materials and instrumentation**

All chemicals including ibuprofen, 1,4–dicarboxylic acid, iron chloride and potassium chloride were commercially purchased from Merck. The D8 Advance Bruker powder diffractometer was used to record the X–ray powder diffraction (XRD) profiles using Cu–Kα beams as excitation sources. The S4800 instrument (Japan) was implemented to capture the scanning electron microscope (SEM) images with the magnification of 7000 using an accelerating voltage source (15 kV). The JEOL JEM 1400 instrument was used to study the transmission electron microscopy (TEM). The characteristics of surface chemistry was investigated by the FT–IR spectra on the Nicolet 6700 spectrophotometer. The N_2_ adsorption/desorption isotherm and pore size distribution data were recorded on the Micromeritics 2020 volumetric adsorption analyzer system. The UV–Vis spectrophotometer was used to determine the ibuprofen concentration at 222 nm. The photoelectron spectrometer Kratos Axis-Ultra was used to recorded the signals of the X-ray photoelectron spectra (XPS) using a monochromatic X-ray source of Al Kα and Casa XPS software was utilized to analyze the XPS spectra. All XPS spectra were calibrated using the C 1s peak (284.8 eV) with a subtraction by Shirley background. Gupta and Sen (GS) multiplets was used to fit the high-spin Fe^3+^ states.

## **Kinetic models**

In kinetic studies, we addressed several models to evaluate the uptake and elucidate the factors affecting adsorption in heterogeneous phase. Therefore, adsorption of ibuprofen drug onto MPC was simulated by kinetics models including pseudo first-order, pseudo second-order, Elovich and intra-particle diffusion equations. By comparing with correlation coefﬁcient R^2^, their fitness was suggested to choose the most compatible model for the description on experimental and calculated data.

Firstly, the pseudo ﬁrst-order model proposed an assumption about the rate of adsorption relating to the number of unabsorbed sites, as presented in Eq. S1 ^1^.

$\log(q_{e}-q_{t})=\log q_{e}-\frac{k_{1}.t}{2.303}$ (S1)

Where, *k*_1_ is the pseudo ﬁrst-order adsorption rate constant (1/min), adsorption capacity *q*_t_ at the time t (min) and equilibrium adsorption capacity *q*_e_ (mg/g) at the equilibrium time (min)

Meanwhile, the pseudo second-order equation (Eq. S2) is used to depict the adsorption via chemisorption pathway with rate constant *k*_2_ (g/mg min) and initial adsorption rate *H* (mg/g min) (Eq. S3) ^2^.

 (S2)

$H=k_{2}. q_{e}^{2}$ (S3)

The Elovich model (Eq. S4) describes the heterogeneous diffusion process of the adsorption of gases on heterogeneous surfaces or liquid/gas phase by the reaction rate and diffusion factor ^3^. The coefficients of Elovich model are defined by the adsorption rate α (mg/g min) and the constant of desorption β (g/mg).

$q_{t}=\frac{1}{\beta}.\ln(\alpha.\beta)+\frac{1}{\beta}.\ln(t)$ (S4)

Finally, intra-particle diffusion mechanism of ibuprofen molecules on MPC at room temperature can be described by Bangham equation (Eq. S5) as follows ^4^:

$\mathrm{loglog} \left(\frac{C_{o}}{C_{o}-q_{t}.\frac{m}{V}}\right)=\log\left(\frac{k_{B}}{2.303.V}\right)+\alpha_{B}.\log(t)$ (S5)

## **Isotherm models**

The isotherm models are commonly utilized to explain the relationship of the adsorbate molecules in liquid/solid phase under the adsorption equilibrium state. Moreover, the range of ibuprofen concentration was experimented from 5 mg/L to 20 mg/L at room temperature. In this study, isotherm equations including Langmuir, Freundlich, Temkin, and Dubinin-Radushkevich (D-R) were investigated.

In detail, the Langmuir equation assumes the monolayer adsorption behavior of ibuprofen molecules on the MPC surface by dynamically balancing the relative rates of adsorption and desorption without lateral interaction of ibuprofen molecules ^5^. This model can be expressed by Eq. S6, giving the isotherm parameters such as equilibrium adsorption capacity Q_e_ (mg/g), equilibrium ibuprofen concentration C_e_ (mg/L), maximum adsorption capacity Q_m_ (mg/g), Langmuir constants K_L_ (L/mg) and R_L_ are defined by Eq. S7. When R_L_ is ranged from 0 and 1, the adsorption of ibuprofen onto materials is considered as a favorable process.

$\frac{1}{Q_{e}}=\left(\frac{1}{Q_{m}K_{L}}.\right)\frac{1}{C_{e}}+\frac{1}{Q_{m}}$ (S6)

$R_{L}=\frac{1}{1+K_{L}C_{o}}$ (S7)

The experimental ibuprofen uptake parameters can be calculated utilizing the Freundlich isotherm, which assumes multilayer adsorption occurring on heterogonous phase surfaces without any uniform distribution of heat of energies ^6^. Values of K_F_ (mg/g)(L/mg)^1/n^ and 1/n are determined from the intercept and slope of the Freundlich equation (Eq. 8). Coefficients such as 1/n and K_F_ (mg/g)(L/mg)^1/n^ are determined from Eq. S8. Note that the magnitude of exponent (1/n) indicates the good favorability of the adsorption of ibuprofen in the range from 0.1 to 0.5 ^7^.

$\ln Q_{e}=\ln K_{F}+\frac{1}{n}\ln C_{e}$ (S8)

$Q_{e}=B_{\text{T}}\ln K_{\text{T}}+B_{\text{T}}\ln C_{e}$ (S9)

$B_{\text{T}}=\frac{RT}{b}$ (S10)

Meanwhile, Temkin isotherm model can be used to find out the effects of indirect interactions of ibuprofen molecules with adsorbent, as shown in Eq. S9 ^8^. Theoretically, B_T_ is a coefficient related to heat of adsorption isotherm, equilibrium adsorption capacity Q_e_ (mg/g) versus logarithm of equilibrium concentration C_e_ (mg/L) is used to determine the constants of K_T_ (L/g) and b (J/mol), and R is the gas constant (8.314 J/mol K) (Eq. S10).

$\ln q_{e}=\ln Q_{m}-B\varepsilon^{2}$ (S11)

$\varepsilon=RT\ln\left(1+\frac{1}{C_{e}}\right)$ (S12)

$E=\frac{1}{\sqrt{2B}}$ (S13)

The Dubinin and Radushkevich (D-R) isotherm (Eq. S11) explains the state of chemical/physical adsorption via the mean energy of adsorption, which its parameters including B (mol^2^/kJ^-2^) and Q_m_ (mg/g) are the activity coefficients, Polanyi potential Ɛ (kJ/mol) and energy of adsorption E (kJ/mol) can be calculated from Eq. S12 and Eq. S13.

## **Thermodynamic study**

The thermodynamic coefficients can be used to well describe the process of the adsorption and the standard parameters could be represented as follows (Eq. S14):

$\Delta G=-RT\ln K_{C}$ (S14)

Where, *K*_C_ is the adsorption equilibrium constant and *T* (K) is temperature at equilibrium point. *K*_C_ is the ratio of equilibrium concentration of adsorbent between liquid and solid phase and can be determined as follows (Eq. S15):

$\ln K_{C}=\frac{C_{A}}{C_{e}}$ (S15)

Where, C_A_ and C_e_ (ppm) are the equilibrium concentrations in aqueous solution onto adsorbent, respectively. Standard enthalpy (ΔH) and entropy (ΔS) can be calculated by Van’t Hoff isotherm equation as follows (Eq. S16):

$\ln K_{C}=\left(\frac{-\Delta H}{R}\right).\frac{1}{T}+\frac{\Delta S}{R}$ (S16)

# **Figure captions**

**Fig. S1.** UV-Vis spectra and structural formula of ibuprofen simulated by molecular dynamics from the Chem3D program after minimizing the energy by MM2 simulation

**Fig. S2**. Nitrogen adsorption/desorption (a,b) and pore distribution (c,d) plots of MIL-53 (Fe) (a,c) and MPC material (c,d)

**Fig. S3.** Kinetic models for adsorption of ibuprofen on MPC

**Fig. S4.** Isotherm models for adsorption of ibuprofen on MPC

**
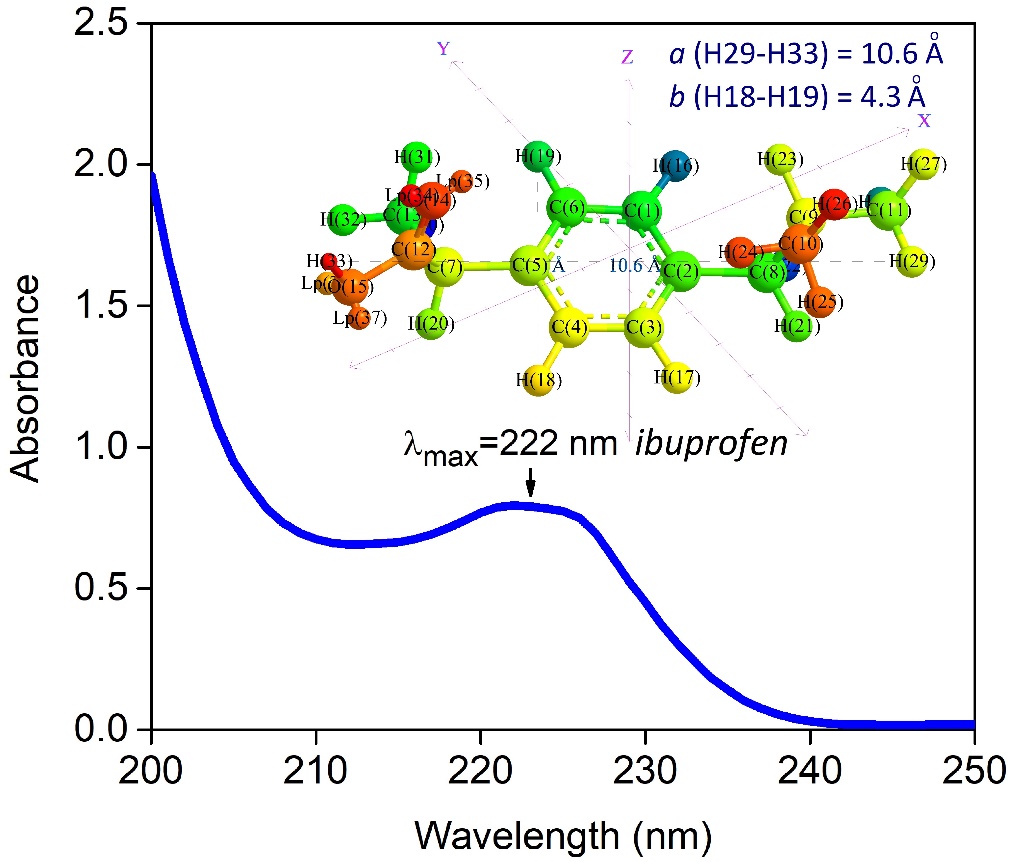
**

**Fig. S1.** UV-Vis spectra and structural formula of ibuprofen simulated by molecular dynamics from the Chem3D program after minimizing the energy by MM2 simulation

**
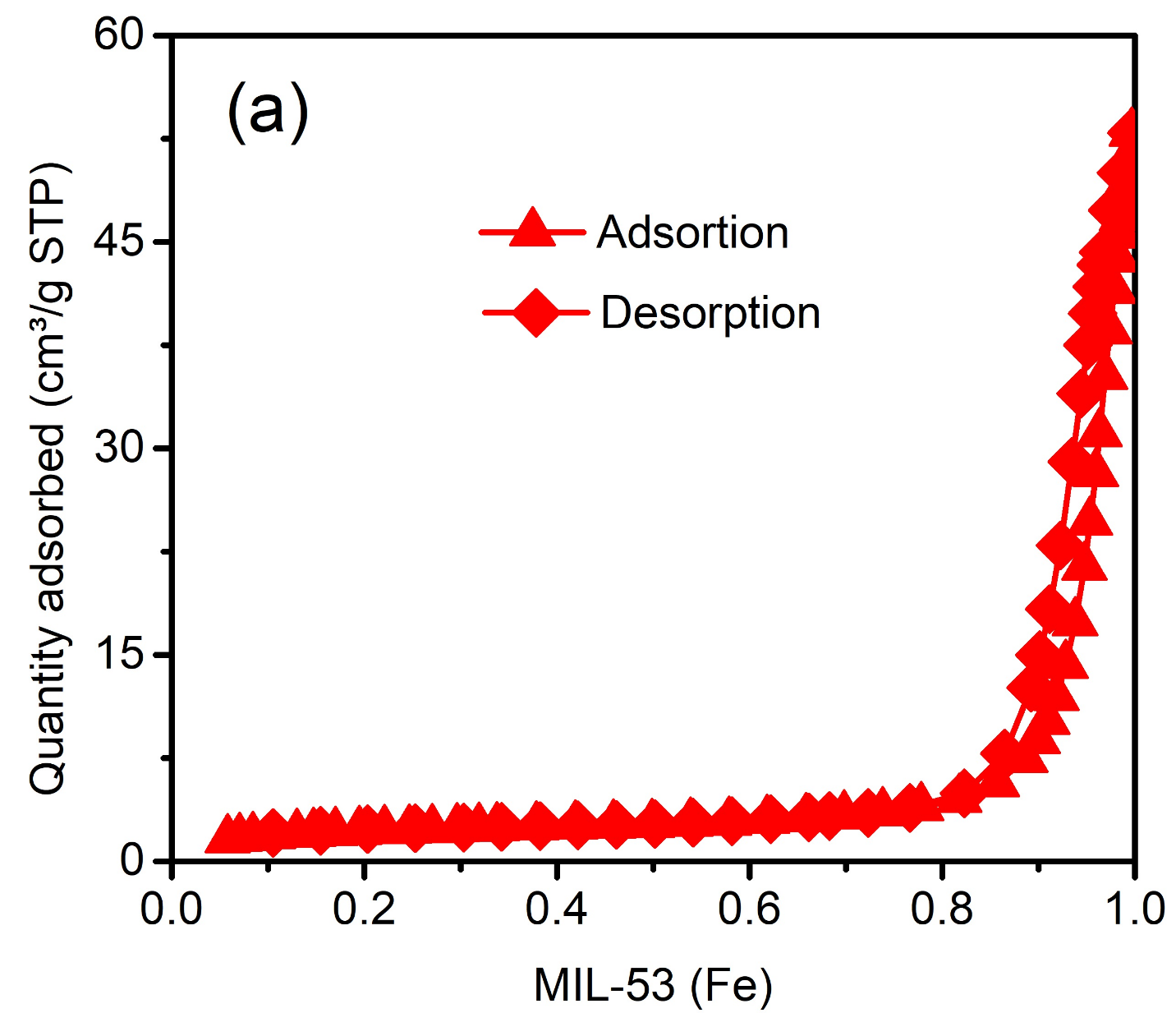
** **
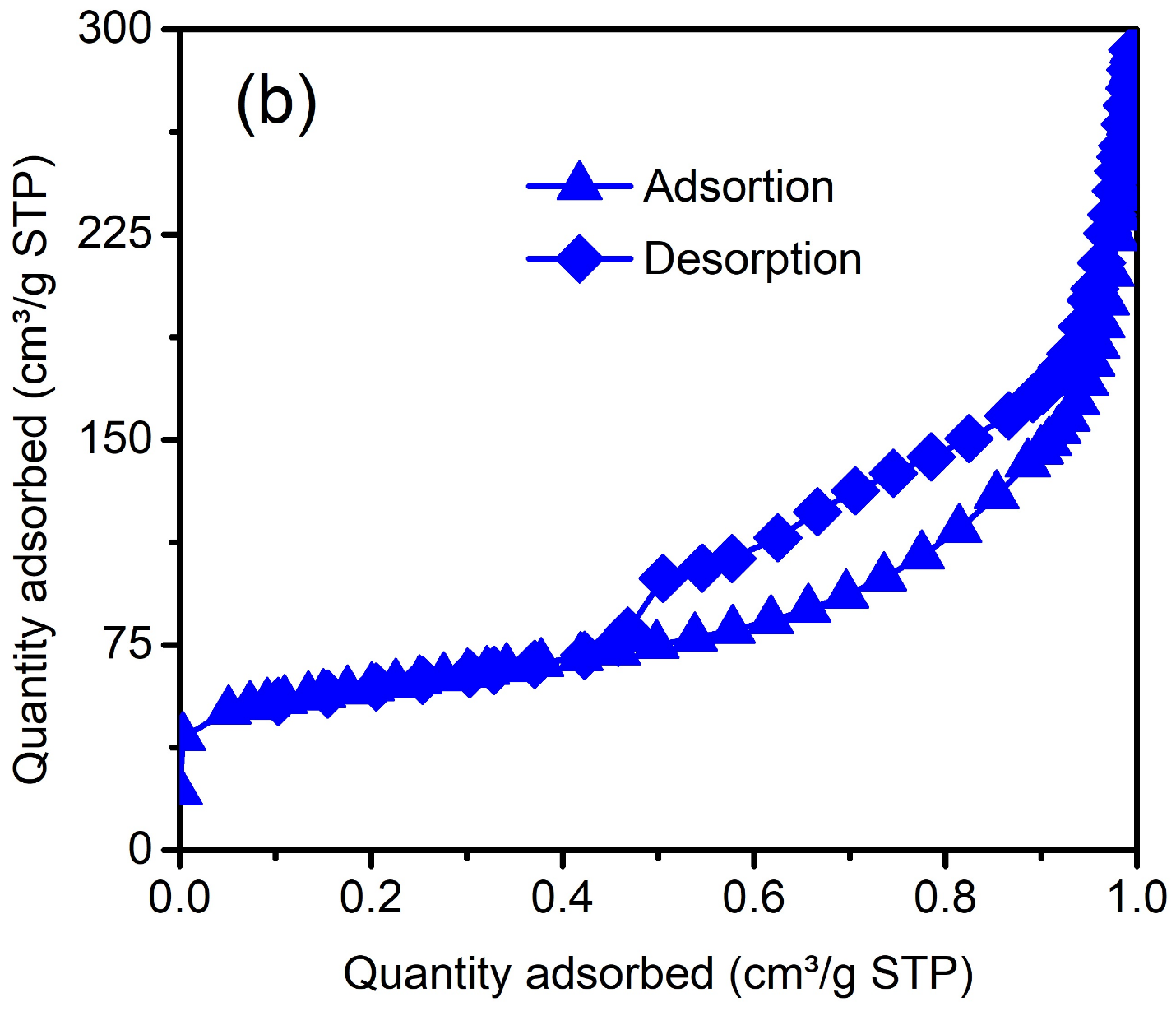
**

**
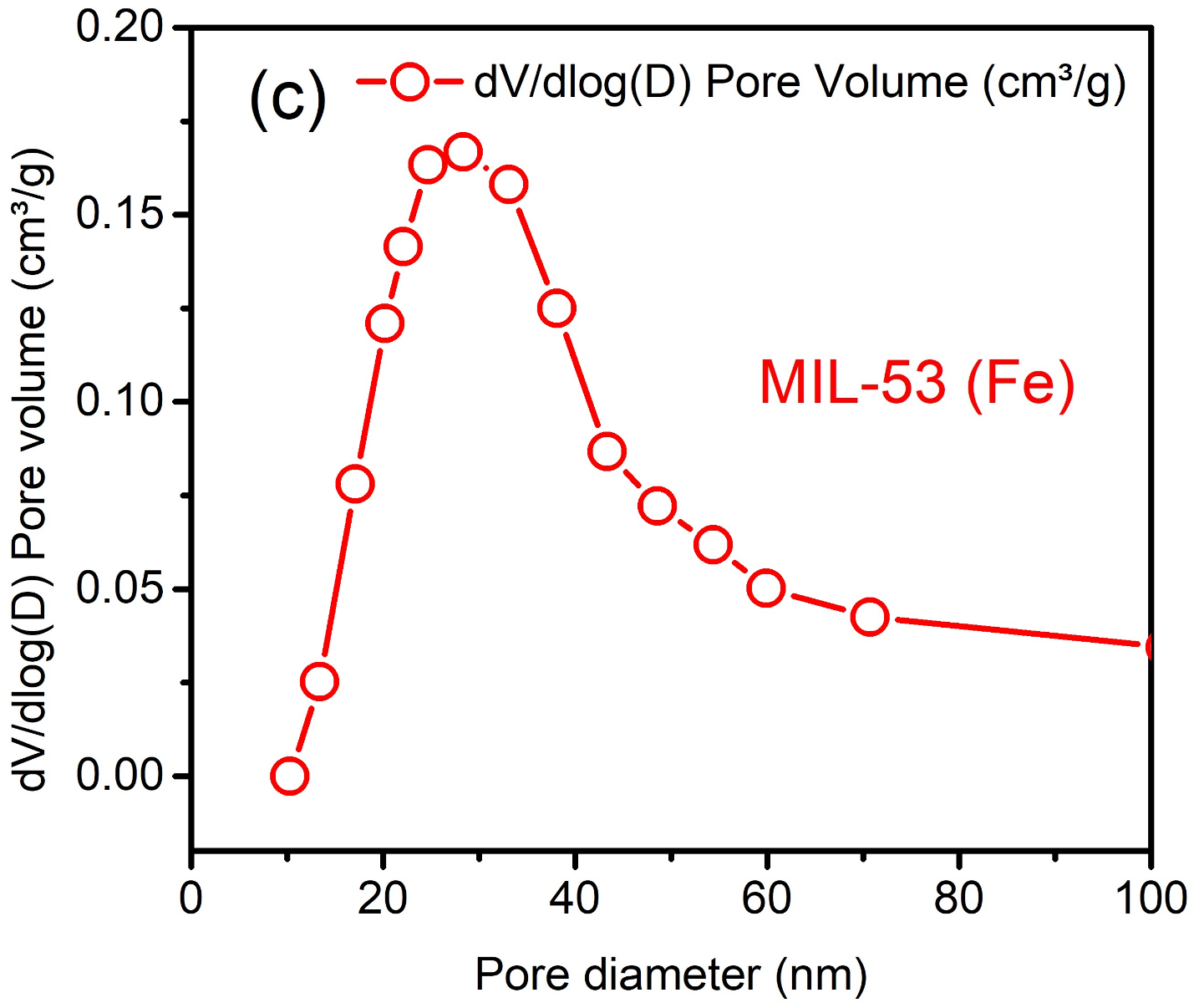
**

**
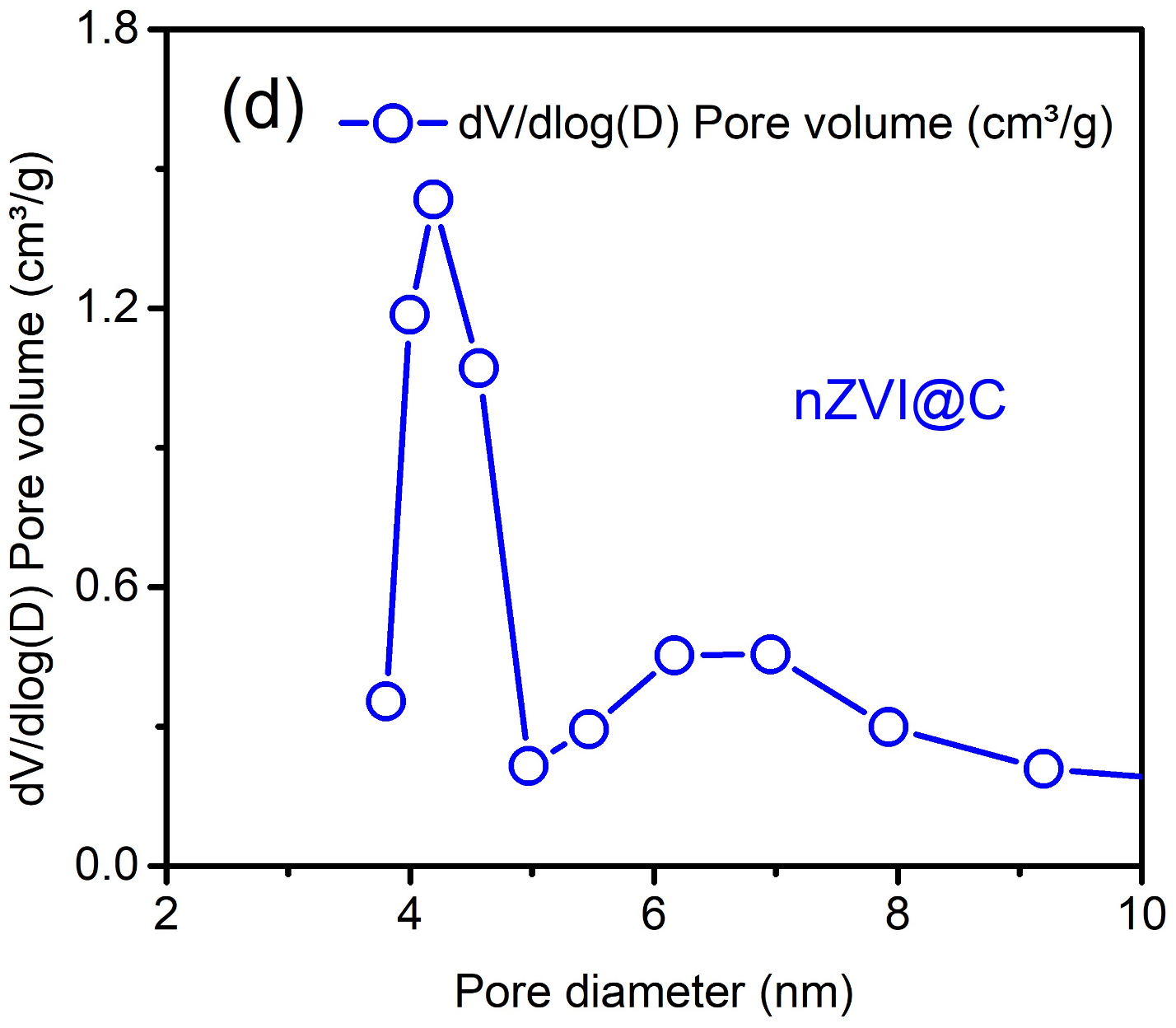
**

**Fig. S2**. Nitrogen adsorption/desorption (a,b) and pore distribution (c,d) plots of MIL-53 (Fe) (a,c) and MPC material (c,d)

**
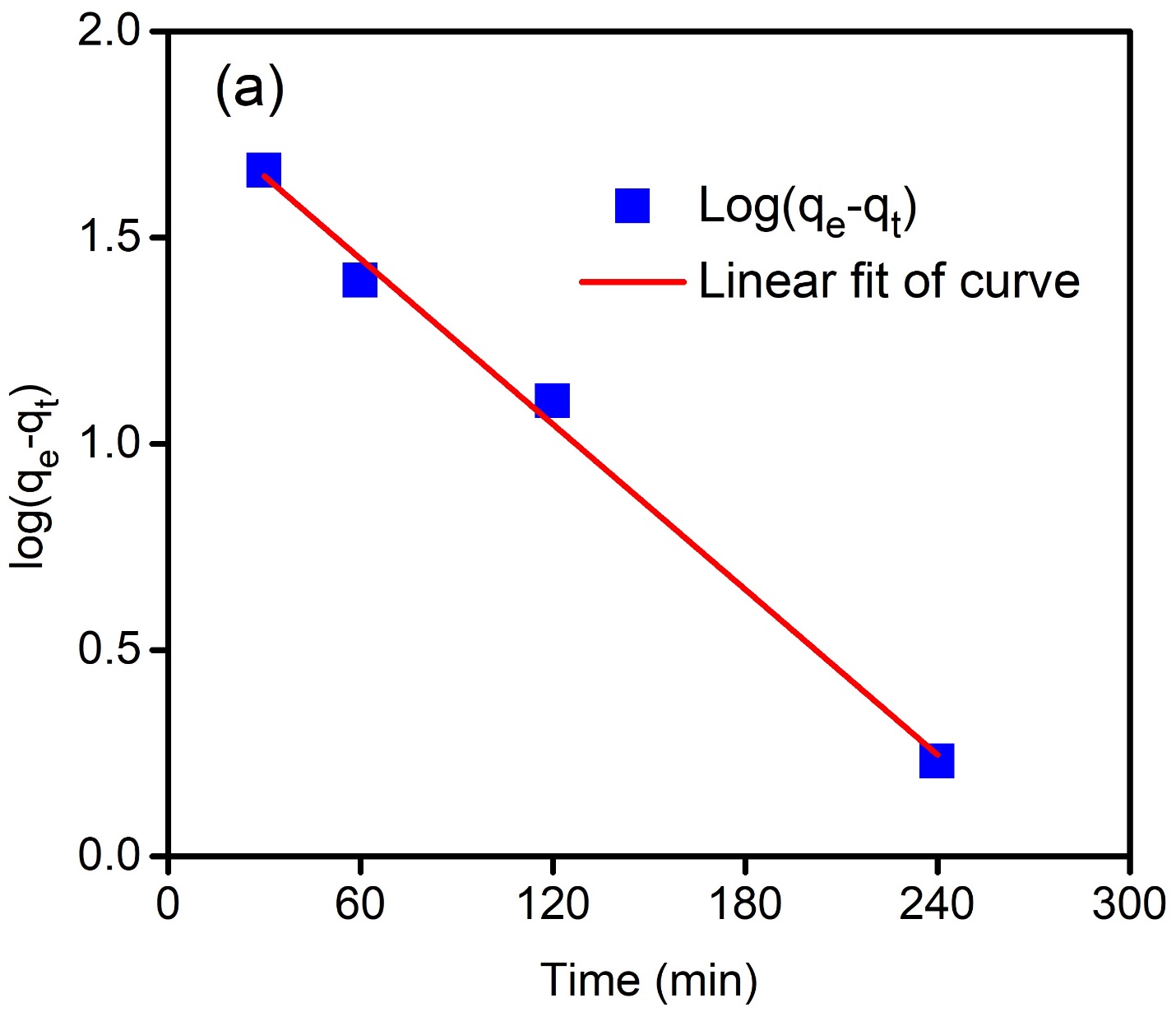
**

**
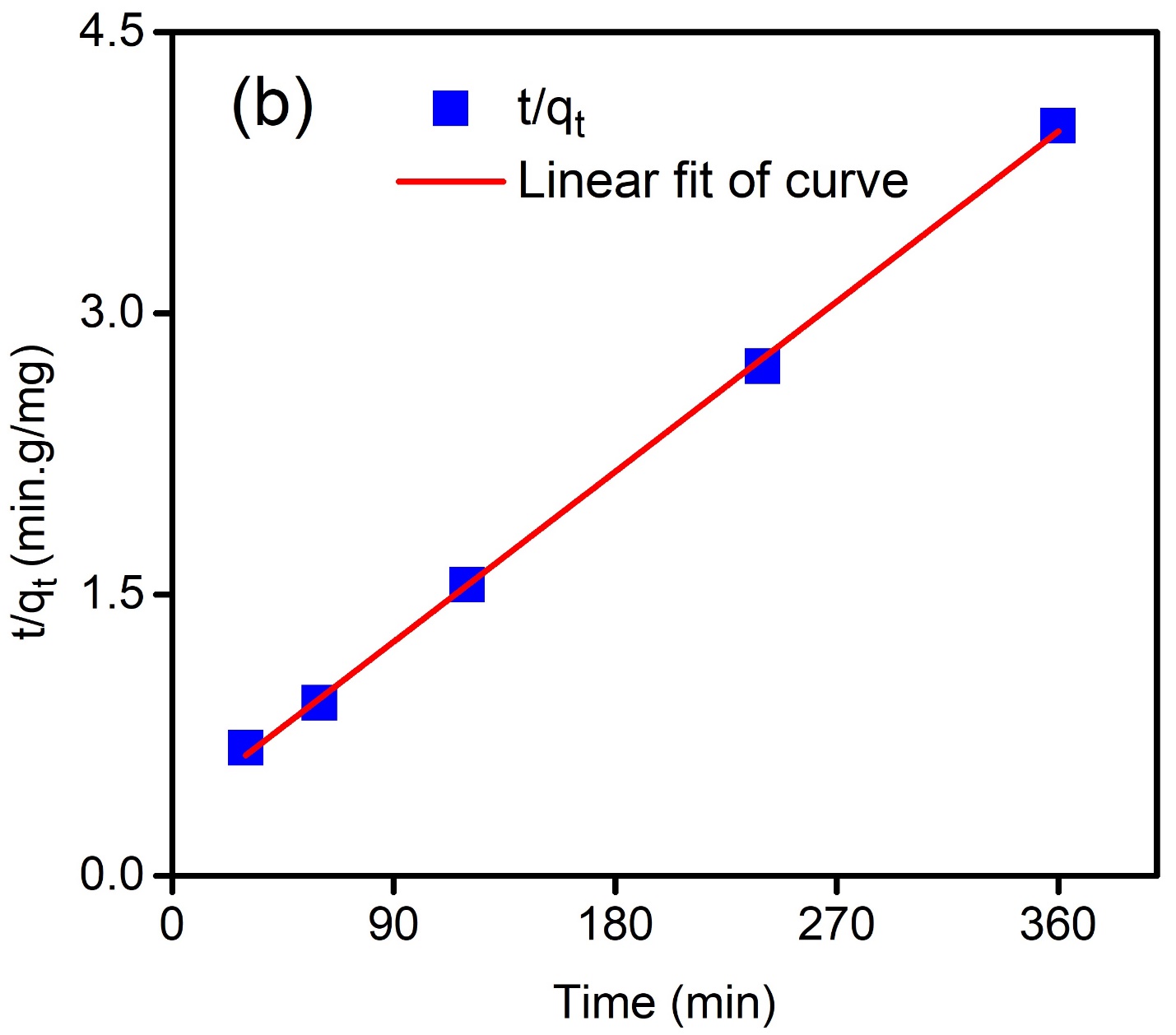
**

**
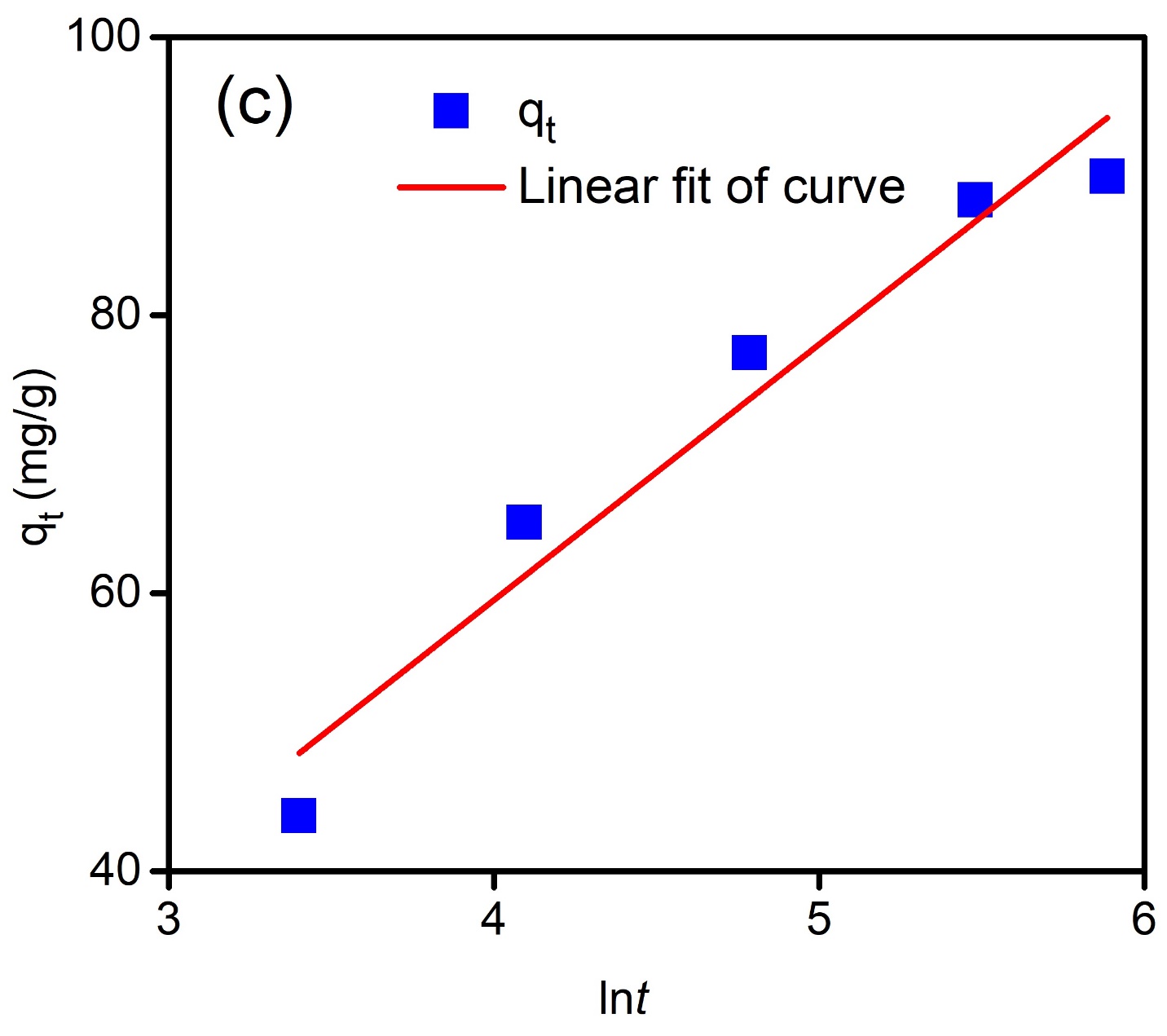
**

**
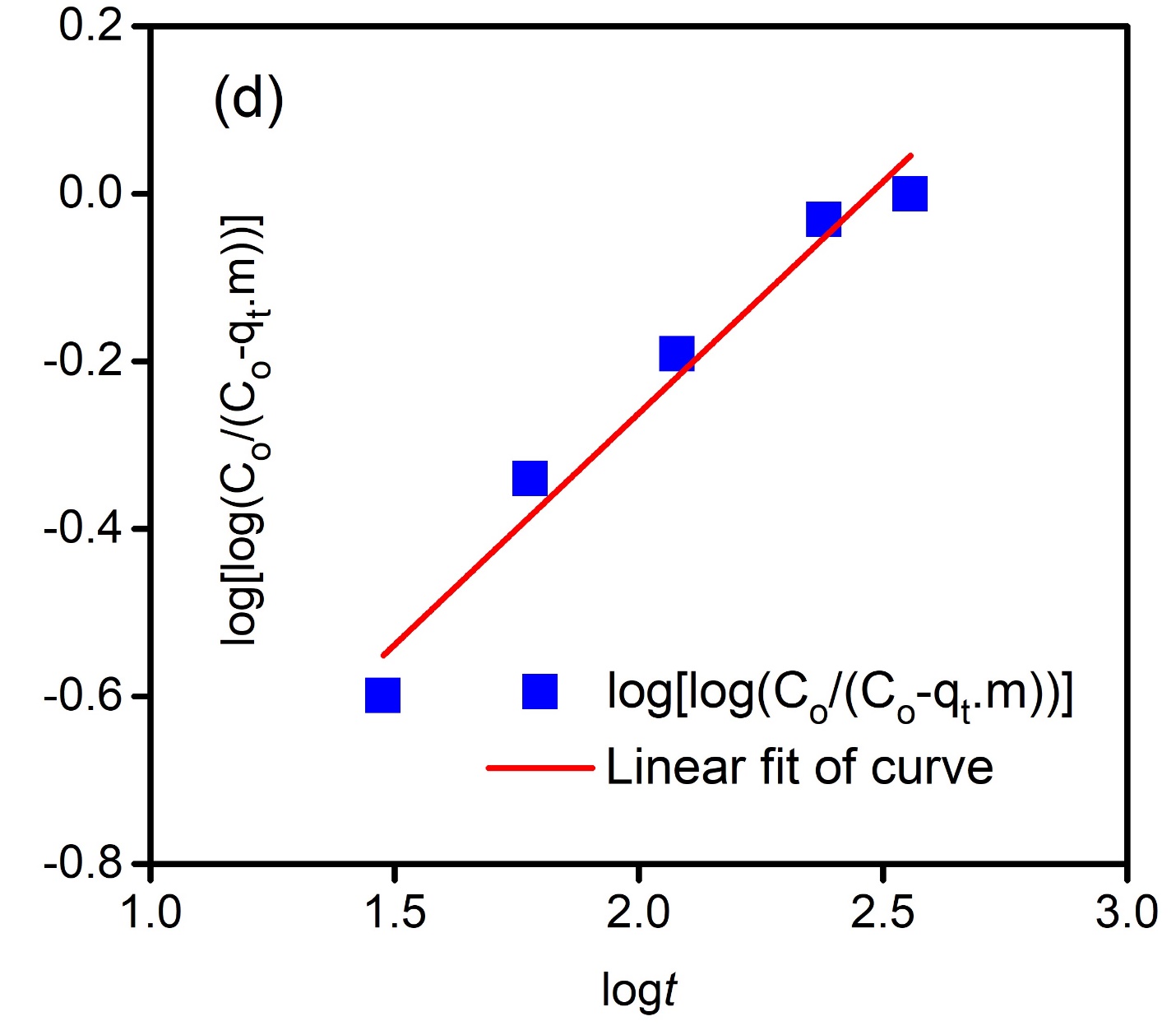
**

**Fig. S3.** Kinetic models for adsorption of ibuprofen on MPC

**
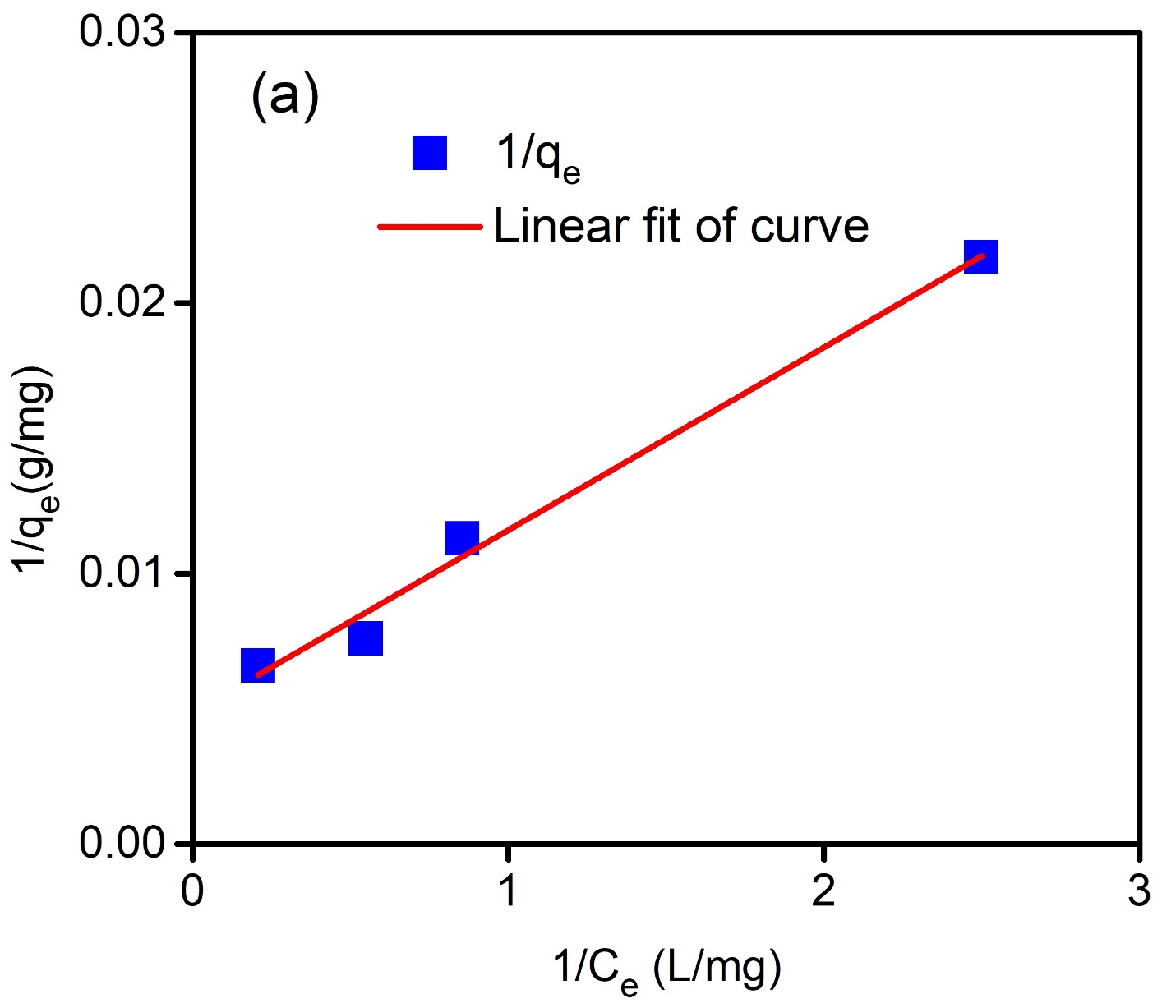
**

**
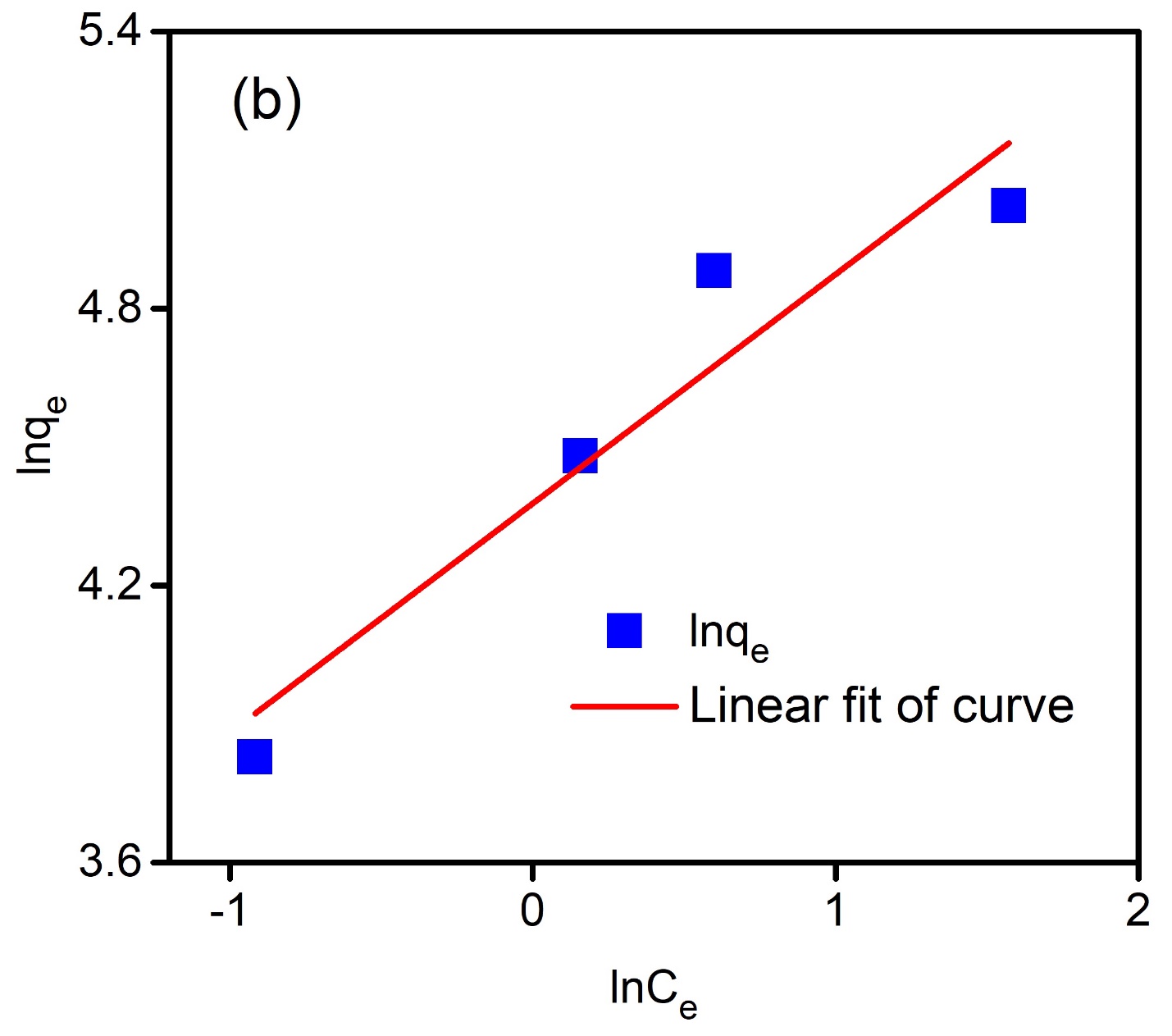
**

**
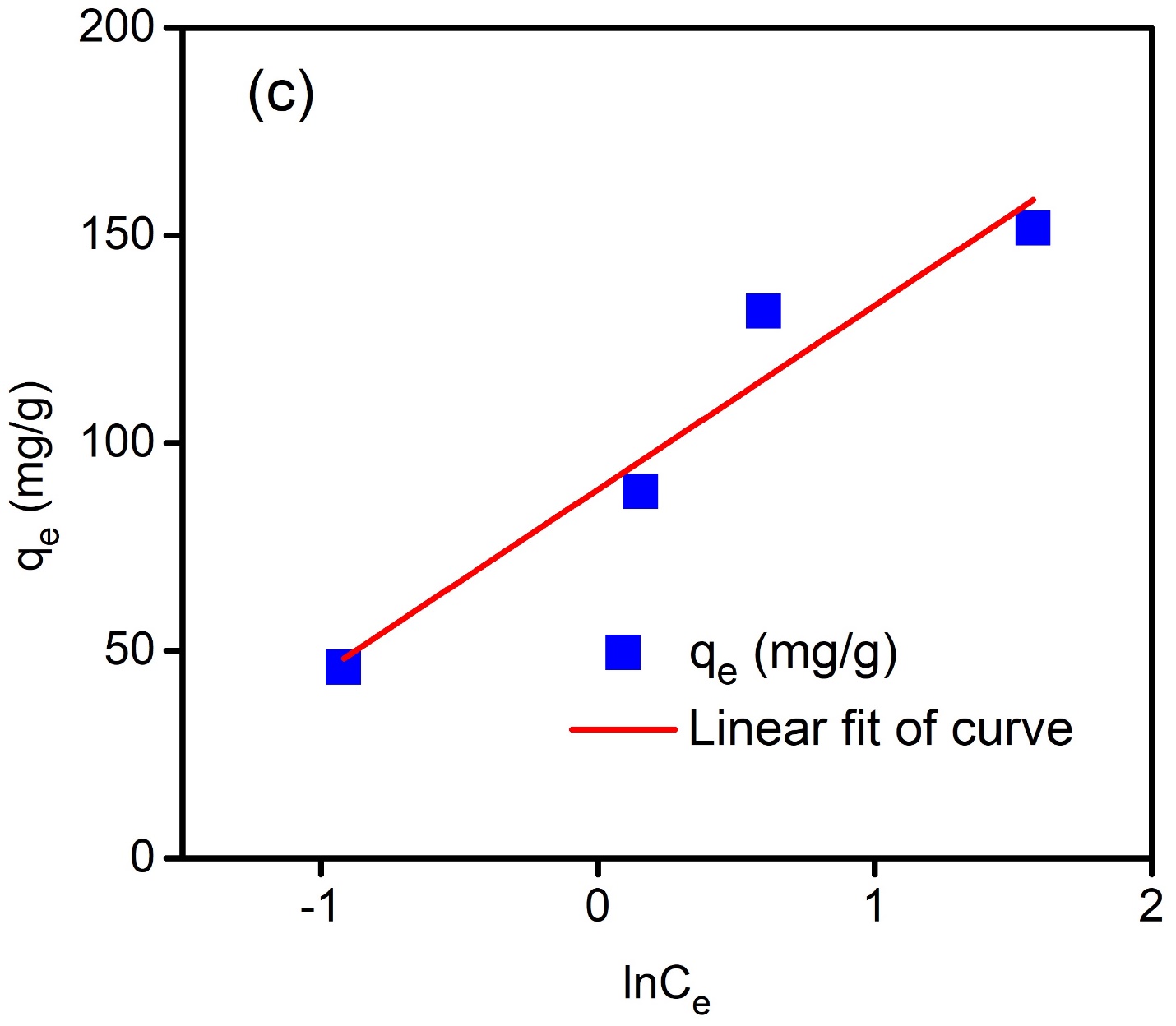
**

**
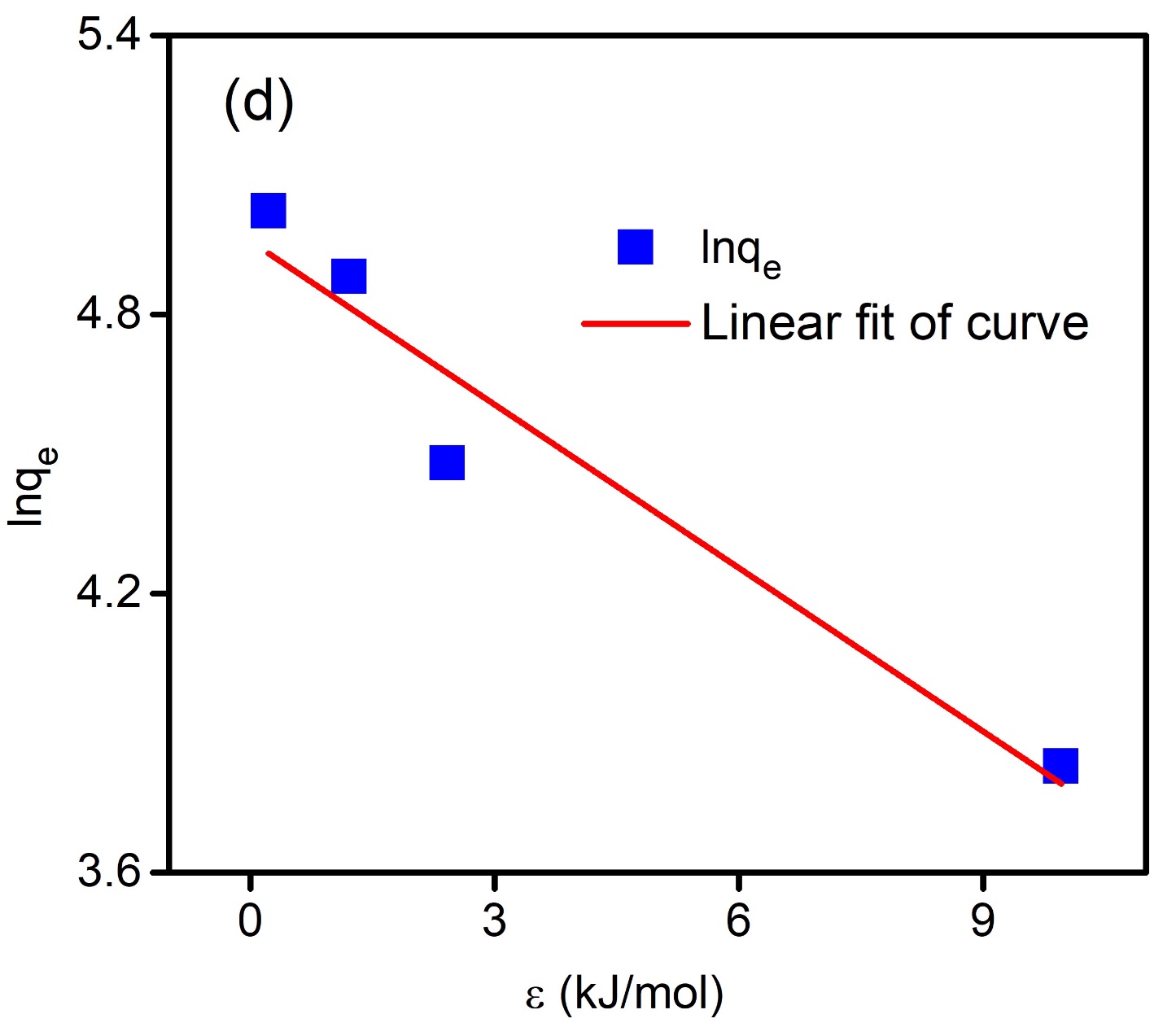
**

**Fig. S4.** Isotherm models for adsorption of ibuprofen on MPC

**Table S1.** Several properties of ibuprofen (CAS No. 15687-27-1)

| Chemical structure | pK_a_ in water (25 ^o^C) ^9^ | Solubility in water (mg/L) ^10^ | Wavelength (nm) | Number of H-acceptor |
| --- | --- | --- | --- | --- |
|  | 5.0 | 21.0 | 222 | 2 |

**Table S2.** Chemical states of Fe, O, C elements and their corresponding parameters

| Sub level | Functional groups | MIL-53 (Fe) | | MPC | |
| --- | --- | --- | --- | --- | --- |
|  |  | Binding energy (eV) | Area (%) | Binding energy (eV) | Area (%) |
| Fe 2p_3/2_ | Fe^2+^ | - | 0 | 708.7, 709.7 | 22.0 |
|  | Fe^3+^ | 710.4, 711.4, 712.3, 713.4 | 100.0 | 710.5, 711.2, 712.0, 713.2, 715.1, 719.4 | 78.0 |
| O 1s | Iron oxides | 530.3 | 6.7 | 530.2 | 16.6 |
|  | C=O | 532.2 | 85.4 | 531.7 | 23.1 |
|  | O-H | 533.9 | 7.9 | - | 0 |
|  | C-O | - | 0 | 533.5 | 31.2 |
|  | O-C=O | - | 0 | 535.0 | 29.1 |
| C 1s | C-C/C=C | 285.0 | 54.8 | 284.3 | 46.1 |
|  | C-O | 285.4 | 23.4 | 285.5 | 39.9 |
|  | C=O | 289.0 | 18.4 | 288.3 | 3.8 |
|  | O-C=O | 291.1 | 3.2 | 290.1 | 10.2 |

## **References**

1 S. Eris and S. Azizian, *J. Mol. Liq.*, 2017, **231**, 523–527.

2 S. Eris and S. Azizian, *Sep. Purif. Technol.*, 2017, **179**, 304–308.

3 D. John Babu, P. King and Y. Prasanna Kumar, *Int. J. Environ. Sci. Technol.*, , DOI:10.1007/s13762-018-1747-2.

4 M. Kavand, N. Asasian, M. Soleimani, T. Kaghazchi and R. Bardestani, *Process Saf. Environ. Prot.*, 2017, **107**, 486–497.

5 T. Van Tran, Q. T. P. Bui, T. D. Nguyen, N. T. H. Le and L. G. Bach, *Adsorpt. Sci. Technol.*, 2017, **35**, 72–85.

6 L. G. Bach, T. Van Tran, T. D. Nguyen, T. Van Pham and S. T. Do, *Res. Chem. Intermed.*, 2018, **44**, 1661–1687.

7 R. M. Ali, H. A. Hamad, M. M. Hussein and G. F. Malash, *Ecol. Eng.*, 2016, **91**, 317–332.

8 A. Zuorro, G. Maffei and R. Lavecchia, *J. Environ. Chem. Eng.*, 2017, **5**, 4121–4127.

9 M. Di Foggia, S. Bonora, A. Tinti and V. Tugnoli, *J. Therm. Anal. Calorim.*, 2017, **127**, 1407–1417.

10 B. N. Bhadra, I. Ahmed, S. Kim and S. H. Jhung, *Chem. Eng. J.*, 2017, **314**, 50–58.

11 H. Guedidi, L. Reinert, J.-M. Lévêque, Y. Soneda, N. Bellakhal and L. Duclaux, *Carbon N. Y.*, 2013, **54**, 432–443.

12 T. X. Bui and H. Choi, *J. Hazard. Mater.*, 2009, **168**, 602–608.

13 A. S. Mestre, J. Pires, J. M. F. Nogueira, J. B. Parra, A. P. Carvalho and C. O. Ania, *Bioresour. Technol.*, 2009, **100**, 1720–1726.

14 H. Mansouri, R. J. Carmona, A. Gomis-Berenguer, S. Souissi-Najar, A. Ouederni and C. O. Ania, *J. Colloid Interface Sci.*, 2015, **449**, 252–260.
